# Genome-scale CRISPR-Cas9 knockout screening in gastrointestinal stromal tumor with Imatinib resistance

**DOI:** 10.1186/s12943-018-0865-2

**Published:** 2018-08-13

**Authors:** Jie Cao, Jianchang Wei, Ping Yang, Tong Zhang, Zhuanpeng Chen, Feng He, Fang Wei, Huacui Chen, He Hu, Junbin Zhong, Zhi Yang, Wensong Cai, Wanglin Li, Qiang Wang

**Affiliations:** Department of General Surgery, Guangzhou Digestive Disease Center, Guangzhou First People’s Hospital, Guangzhou Medical University, the Second Affiliated Hospital of South China University of Technology, 1 Panfu Road, Guangzhou, 510180 Guangdong China

**Keywords:** Gastrointestinal stromal tumor, Imatinib resistance, Genome-scale CRISPR-Cas9 knockout screening

## Abstract

Genome-scale CRISPR-Cas9 Knockout Screening was applied to investigate novel targets in imatinib-resistant gastrointestinal stromal tumor (GIST). 20 genes and 2 miRNAs have been selected by total reads of sgRNA and sgRNA diversity, which has been further validated in imatinib-resistant GIST cells by CCK8 and qPCR analysis. Our study has finally revealed 9 genes (DBP, NR3C1, TCF12, TP53, ZNF12, SOCS6, ZFP36, ACYP1, and DRD1) involved in imatinib-resistant GIST-T1 cells. TP53 and SOCS6 may be the most promising candidate genes for imatinib-resistance due to the possible signaling pathway, such as apoptosis pathway and Wnt signaling pathway, JAK-STAT signaling pathway. It is necessary to perform more studies to discover novel targets in imatinib-resistant GIST, including DBP, NR3C1, TCF12, ZNF12, ZFP36, ACYP1 and DRD1.

**Electronic supplementary material:**

The online version of this article (10.1186/s12943-018-0865-2) contains supplementary material, which is available to authorized users.

Gastrointestinal Stromal Tumor (GIST) is the most frequent mesenchymal tumor in the gastrointestinal tract [1]. The activating mutations in PDGFRA or KIT are observed in GIST, which are the key molecular drivers in tumor pathogenesis [2]. Imatinib mesylate, also known as Glivec®, is used as tyrosine kinase inhibitor for standard targeted therapy in GIST. However, secondary resistance to imatinib with disease progression is observed in about half of patients in 2 years of therapy. The mechanisms of imatinib-resistance in GIST have been validated in some extent, such as PI3K/AKT/mTOR pathway [3]. Due to the complexity of imatinib-resistant mechanisms, it is necessary to discover novel targets to imatinib-resistant in GIST. The RNA-guided CRISPR-associated nuclease Cas9 is an effective method to introduce targeted loss-of-function mutations at the specific sites in genome with low noise, consistent activity across reagents and minimal off-target effects [4]. This system has been previously reported to identify drug resistant genes with high efficiency in vitro [5]. Here, we sought to identify novel genes which are critically important to imatinib-resistance in GIST by genome-scale CRISPR-Cas9 knockout screening.

## Genome-scale CRISPR-Cas9 knockout screening for imatinib resistance

**Fig. 1:**
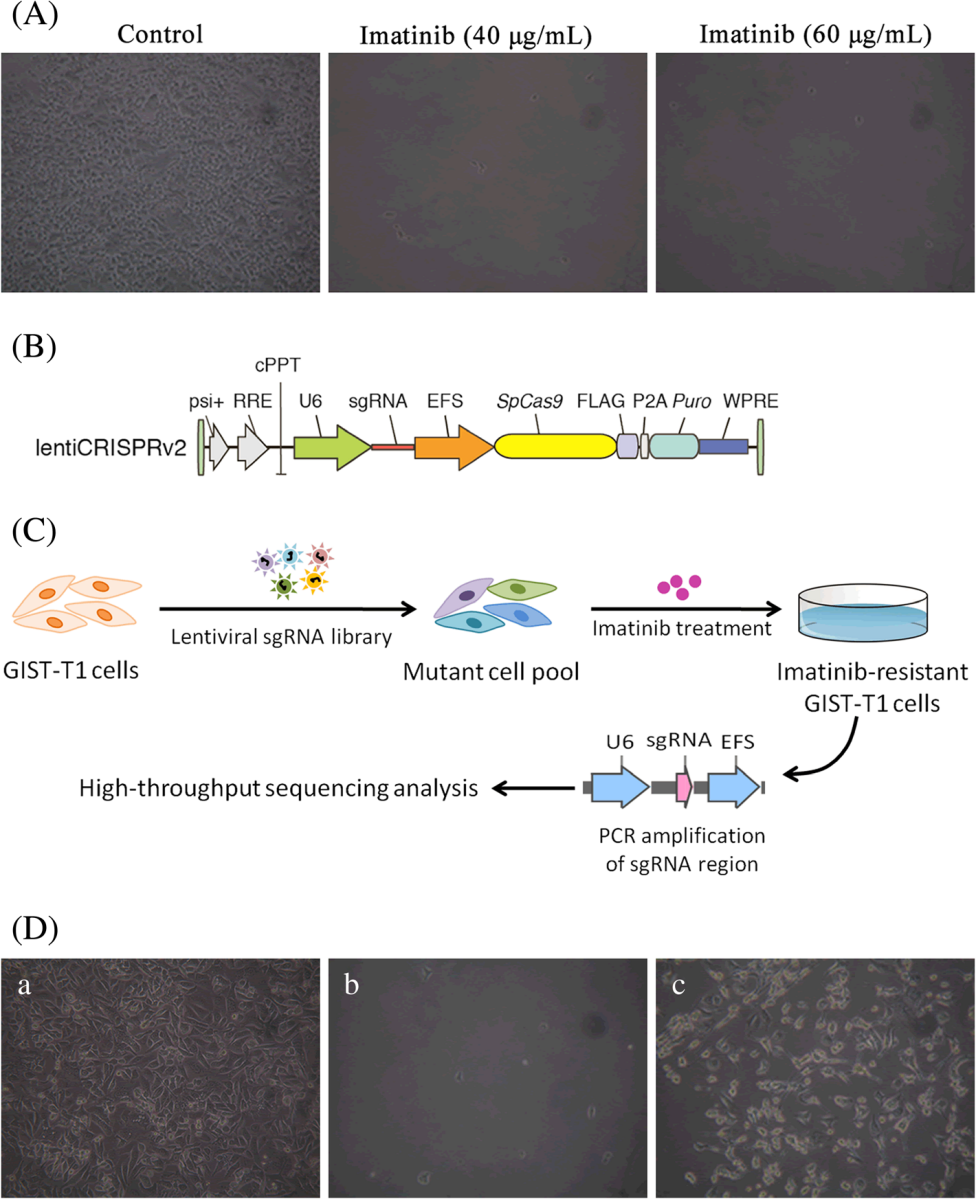
Schematic and results of functional screening by sgRNA library and imatinib treatment. **a** Optical microscopic images of GIST-T1 cells treated with imatinib at day 4. **b** Structure of one vector lentiviralGeCKO system; **c** Schematic of imatinib-resistant GIST-T1 cells construction for high-throughput sequencing analysis; **d** Optical microscopic images of GIST-T1cells transfected with lentiviral sgRNA library and treated with imatinib. a-control, b-imatinib (40 μg/mL), c-transfection and imatinib (40 μg/mL)

To determine the minimum lethal dose (MLD) of imatinib in human GIST-derived cell line GIST-T1 cells, different concentrations of imatinib were added into GIST-T1 cells (results shown in Additional file 1: Figure S1). As shown in Fig. 1a, the cell number in control group was far more than that in imatinib groups (40 μg/mL) at day 4. 40 μg/mL was considered as the MLD of imatinib in GIST-T1 cells, which would be used in the following experiments.

To identify genes related to imatinib drug resistance in GIST, genome-scale CRISPR-Cas9 knockout screening was performed in the GIST-T1 cells. We applied a human genome-scale CRISPR knockout library A (hGeCKOa) and B (hGeCKOb) to transfect GIST-T1 cells separately. hGeCKOa and hGeCKOb contain 65,383 and 58,028 sgRNAs targeting 19,050 genes, respectively (see Additional file 2: Table S1 and Additional file 3: Table S2). A single vector lentiviralGeCKO system (lentiCRISPRv2, Fig. 1b) was used to deliver sgRNA, Cas9 and puromycin selection marker into GIST-T1 cells. Figure 1c displayed a schematic of imatinib-resistant GIST-T1 cells construction for high-throughput sequencing analysis. After imatinib treatment, the genomic DNA of surviving cells (imatinib-resistant GLIST-T1 cells) was extracted for PCR amplification of sgRNA-coding region, followed by high-throughput sequencing analysis. As shown in Fig. 1d, most of GIST-T1cells transfected with lentiviral sgRNA library were survived at day 6 after imatinib treatment, indicating that imatinib-resistant GIST-T1 cells were obtained. The successful transfection of lentiCRISPRv2 vector in GIST-T1 cells was confirmed by electrophoresis (results shown in Additional file 4: Figure S2A). After exposure to imatinib, a small group of GIST-T1 cells transfected with hGeCKOa or hGeCKOb was rendered drug resistance to imatinib, which was cultured and collected to extract DNA for PCR amplification (as shown in Additional file 1: Figure S2B-C).

## Enriched sgRNAs in GIST-T1 cells with imatinib resistance

**Fig. 2:**
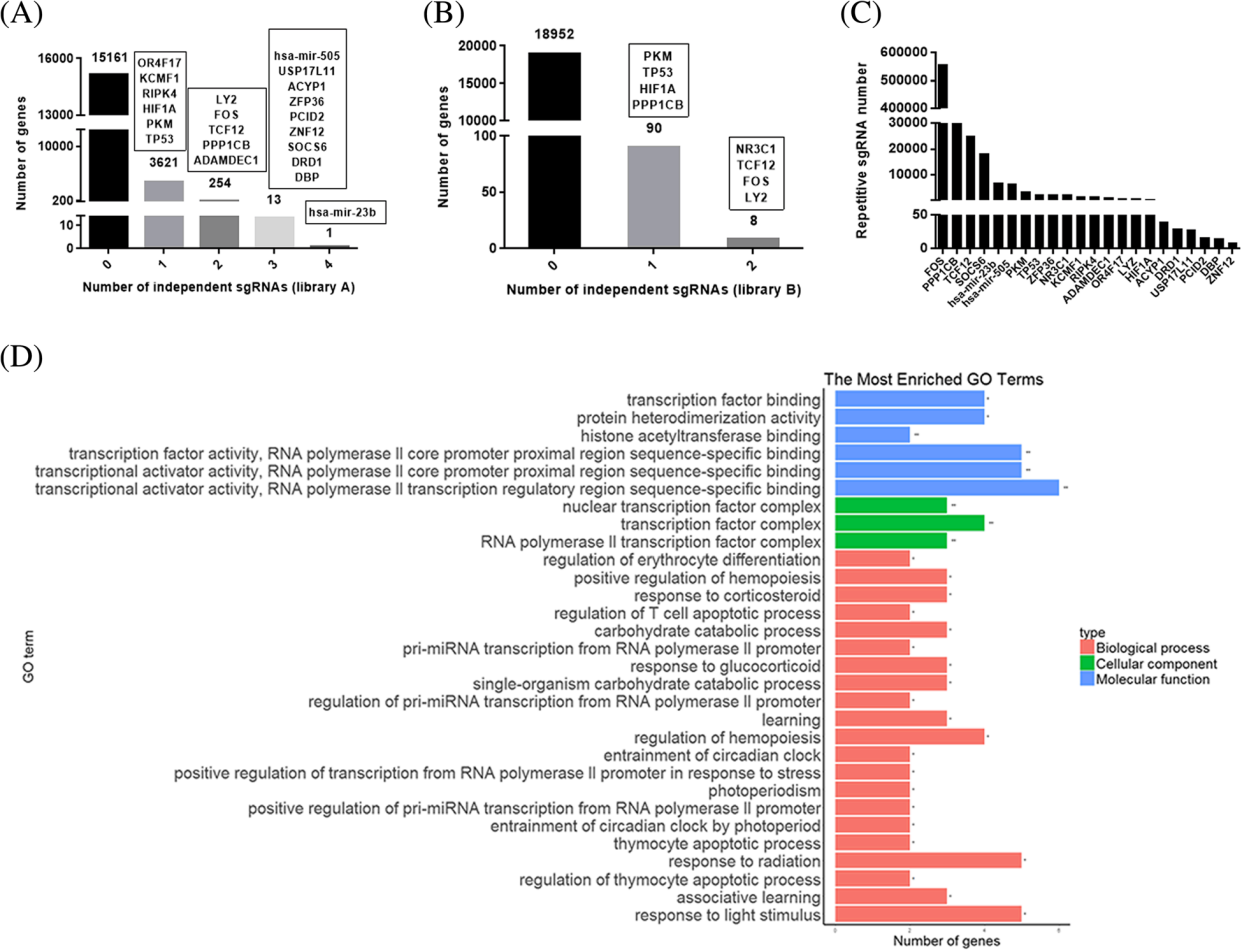
GeCKO screening in GIST-T1 cells reveals genes and miRNAs whose loss confers imatinib resistance. **a** & **b** Number of genes/miRNA with 0, 1, 2, 3 or 4 significantly enriched hGeCKOa (**a**) and hGeCKOb (**b**) sgRNAs targeting that genes/miRNA. Genes/miRNAs were selected by total reads of sgRNA and sgRNA diversity, as shown in black boxes. **c** Total reads of sgRNA for selected genes/miRNAs. **d** Gene ontology of candidate genes

The results of high-throuoghput sequencing analysis have been shown in Additional file 5: Table S3. For a subset of genes/miRNA, we found enrichment of sgRNAs targeting each gene after imatinib treatment (Fig. 2a, b), suggesting that loss of these particular genes contributes to imatinib resistance. For further study, we have chosen 20 genes and 2 miRNAs by the total reads of sgRNAs and sgRNA diversity, as listed in Additional file 6: Table S4. The total reads of sgRNAs for selective genes/miRNAs were also shown in Fig. 2c. Gene ontology (GO) analysis was performed for the selected 20 genes to analyze their roles in biological process, molecular function and cellular component. Top 30 pathways from GO analysis of the 20 genes were shown in Fig. 2d. Detailed information of GO analysis for these genes was listed in Additional file 7: Table S5.

## GeCKO screening results of imatinib in GIST cells

**Fig. 3:**
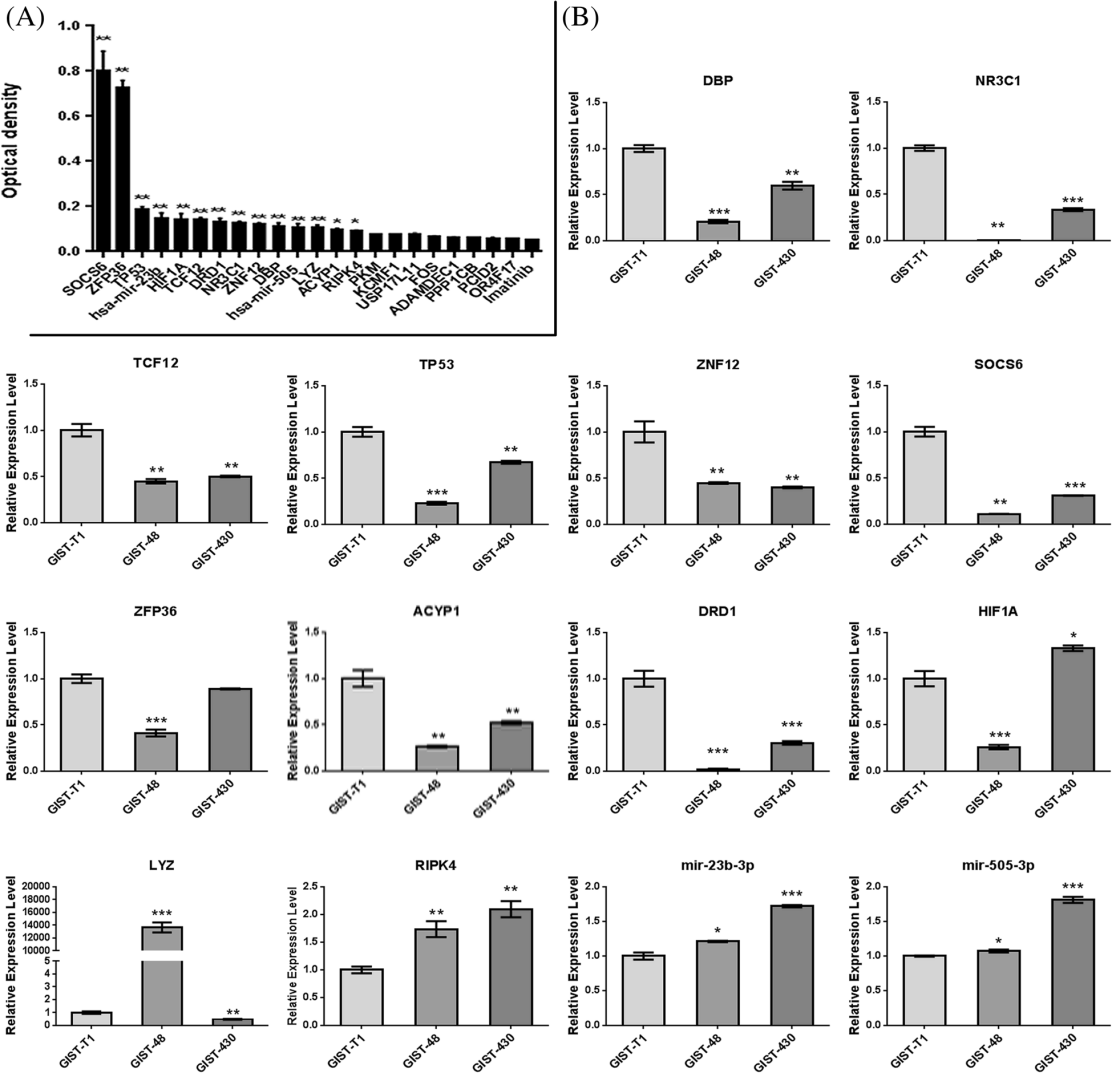
GeCKO screening results of imatinib in GIST cells. **a** CCK8 assay for GIST-T1 cells with individual gene or miRNA knockouts; **b** qPCR analysis for 12 genes and 2 miRNAs in GIST-T1 cells (imatinib-sensitive), imatinib-resistant GIST-48 and imatinib-resistant GIST-430 cells

To validate the function of candidate genes/miRNA identified from GeCKO screen, we generated individual gene or miRNA knockouts in GIST-T1 cells. We tested their susceptibility to imatinib in GIST-T1 cells by using CCK8 assays. As shown in Fig. 3a, there were 12 genes and 2 miRNAs being resistant to imatinib, especially SOCS6 and ZFP36. The majority of these resistant genes/miRNAs were targeted by two or more independent sgRNAs, indicating the tight relative between sgRNA diversity and resistant genes/miRNA. Optical microscopic images of GIST-T1cells with individual gene/miRNA knockouts have been shown in Additional file 8: Figure S3.

Furthermore, qPCR was applied to determine the expression levels of 12 genes and 2 miRNAs in GIST-T1 cells (imatinib-sensitive), imatinib-resistant GIST-48 and imatinib-resistant GIST-430 cells with the aim of confirming their functions. As shown in Fig. 3(b), the expression levels of 9 genes were significantly lower in imatinib-resistant GIST-48 and imatinib-resistant GIST-430 cells than that in GIST-T1 cells, including SOCS6, ZFP36, DBP, NR3C1, TCF12, TP53, ZNF12, ACYP1, and DRD1. These 9 genes (DBP, NR3C1, TCF12, TP53, ZNF12, SOCS6, ZFP36, ACYP1, and DRD1) might be the potential genes for imatinib-resistance, while HIF1A, LYZ, mir-23b-3p and mir-505-3p showed uncertain relationship with imatinib-resistance.

It is worth mentioning that these resistant genes included previously reported genes of TP53 in imatinib resistance [6]. In addition, reported genes/miRNAs involved in other drug resistance were also detected, such as SOCS6 [7], ZFP36 [8], and NR3C1 [9]. Except for these reported genes in drug resistance, we have revealed novel genes involved in imatinib intoxication mechanisms, including TCF12, DRD1, ZNF12, DBP, and ACYP1.

Fas-mediated apoptosis pathway and the loss of TP53 were associated with imatinib resistance in chronic myeloid leukemia, indicating that the mechanism of action of imatinib is possibly responsible for imatinib resistance in cancers [6, 10]. Similarly, the loss of TP53 in GIST-T1 cells showed obvious imatinib resistance in our study, which was consistent with the reported study. The induction of SOCS6 [7] and ZFP36 [8] may contribute to drug resistance in breast cancer and omental adipose tissue, respectively. In contrast, the loss of SOCS6 and ZFP36 in GIST-T1 cells resulted in imatinib resistance. Further investigation should be performed to validate the function and mechanisms of SOCS6 and ZFP36 in imatinib resistance. The KEGG pathway analysis of candidate genes has been listed in Additional file 9: Table S6. Additional file 10: Figure S4A has shown the potential signaling pathway contributed to imatinib resistance in GIST, including apoptosis pathway and Wnt signaling pathway, JAK-STAT signaling pathway, which may be related with the loss of TP53 and SOCS6 respectively. More targets to imatinib-resistance would be identified from these candidate genes in the following study. Protein-protein interaction (PPI) network of these candidate genes has been shown in Additional file 10: Figure S4B, which would provide a reference for the discovery of potential targets in imatinib-resistant GIST.

## Conclusions

Our study has validated 9 genes involved in imatinib-resistant GIST-T1 cells by genome-scale CRISPR-Cas9 knockout screening. TP53 and SOCS6 may be the most promising candidate gene for imatinib-resistance due to the possible signaling pathway, such as apoptosis pathway and Wnt signaling pathway, JAK-STAT signaling pathway. It is necessary to perform more studies to discover novel targets in imatinib-resistant GIST, including DBP, NR3C1, TCF12, ZNF12, ZFP36, ACYP1 and DRD1.

## Additional files

Additional file 1: **Figure S1.** Optical microscopic images of GIST-T1 cells treated with imatinib (40, 60 and 80 μg/mL) with observation at day 2, 4 and 5. (TIF 314 kb)

Additional file 2: **Table S1.** human geckov2 library a. (XLSX 2104 kb)

Additional file 3: **Table S2.** human geckov2 library b. (XLSX 1864 kb)

Additional file 4: **Figure S2.** PCR amplification of sgRNA region for deep-sequencing analysis. (A) Successful transfection of lentiCRISPRv2 vector in GIST-T1 cells, as indicated by electrophoresis of the PCR amplification region (192 bp) from lentiCRISPRv2 vector. M: 2000 bp DNA marker; 1: PCR amplification products of lentiCRISPRv2 vector transfected with GIST-T1 cells; 2: PCR amplification products of GIST-T1 cells without lentiCRISPRv2 vector transfection. (B) PCR amplification of sgRNA region from the sgRNA library for deep-sequencing analysis, as indicated by electrophoresis. M: 2000 bp DNA marker. (C) Sequence of sgRNA region (642 bp) for PCR amplification. The black part: linker adaptor; The red part: variable sequence (24 bp) for sequencing analysis. (TIF 81 kb)

Additional file 5: **Table S3.** The results of high-throughput sequencing analysis. (XLSX 263 kb)

Additional file 6: **Table S4.** Candidate genesmiRNAs with sgRNA sequence, total reads and diversity. (DOCX 13 kb)

Additional file 7: **Table S5.** Detailed information of GO analysis for the selected 20 genes. (DOCX 13 kb)

Additional file 8: **Figure S3.** Optical microscopic images of GIST-T1 cells with individual gene/miRNA knockouts and imatinib treatment. (TIF 606 kb)

Additional file 9: **Table S6.** KEGG pathway analysis of candidate genes. (DOCX 14 kb)

Additional file 10: **Figure S4.** (A)The potential signaling pathway contributed to imatinib resistance in GIST. The green boxes and the solid arrows represented the previously reported signaling pathways related with imatinib resistance; the orange boxes and the dotted arrows represented the potential signaling pathway contributed to imatinib resistance in GIST. (B) Validated genes (9 genes) in protein-protein interaction network. (TIF 319 kb)

## Abbreviations

ACYP1: Acylphosphatase
AKT: Protein kinase B
CRISPR: Clustered regularly interspaced short palindrome repeats
DBP: D-box binding PAR bZIP transcription factor
DRD1: Dopamine receptor D1
GIST: Gastrointestinal stromal tumor
HIF1A: Hypoxia inducible factor 1 alpha subunit
KEGG: Kyoto encyclopedia of genes and genomes
MLD: Minimum lethal dose
mTOR: Mammalian target of the rapamycin
NR3C1: Nuclear receptor subfamily 3 group C member 1
PCR: Polymerase chain reaction
PDGFRA: Platelet-derived growth factor receptor A kinase
PI3K: Phosphatidylinositol-3-kinase
qPCR: Real-time quantitative qPCR
RIPK4: Receptor interacting serine/threonine kinase 4
SOCS6: Suppressor of cytokine signaling 6
TCF12: Transcription factor 12
TP53: Tumor protein p53
ZFP36: ZFP36 ring finger protein
ZNF12: Zinc finger protein 12
