# Supplementary material for: Genome-scale CRISPR-Cas9 knockout screening in gastrointestinal stromal tumor with Imatinib resistance
Source: Mol Cancer. 2018 Aug 13;17:121. doi: 10.1186/s12943-018-0865-2 (PMC6090611; doi:10.1186/s12943-018-0865-2)
Supplement: Supplementary file 6 — Table S4. Candidate genesmiRNAs with sgRNA sequence, total reads and diversity. (DOCX 13 kb) [file 12943_2018_865_MOESM6_ESM.docx]

Table S4.Candidate genes/miRNAs with sgRNA sequence, total reads and diversity

| Number | Gene symbol or miRNA | Sequence of sgRNA | Total reads of sgRNAs | sgRNA diversity |
| --- | --- | --- | --- | --- |
| 1 | FOS | CTGCAGCCAAATGCCGCAAC | 552746 | 2 |
| 2 | PPP1CB | TCAGTACGAGGATGTCGTCC | 52023 | 2 |
| 3 | TCF12 | TGTTGGGGACAAGCCTTCAT | 24460 | 2 |
| 4 | SOCS6 | ACGAGCGCACCTGCATGAAC | 17656 | 3 |
| 5 | hsa-mir-23b | AAGATTAAAATCACATTGCC | 6596 | 4 |
| 6 | hsa-mir-505 | ACACTTGCTGGTTTCCTCTC | 5955 | 3 |
| 7 | PKM | CAGCCACGTACCAACATTCA | 2940 | 1 |
| 8 | TP53 | CCGGTTCATGCCGCCCATGC | 2013 | 1 |
| 9 | ZFP36 | GTGCCCGTGCCATCCGACCA | 1904 | 3 |
| 10 | NR3C1 | AGTACTGCAGTAGGGTCATT | 1768 | 2 |
| 11 | KCMF1 | CCAATATGTGCAGCGTTACC | 1254 | 1 |
| 12 | RIPK4 | CTCACCTGTCGTCGACGTGC | 1103 | 1 |
| 13 | ADAMDEC1 | TCGGACCCTACCATGTTCTC | 865 | 2 |
| 14 | OR4F17 | GCCGTGCACTTACCCTTCTG | 593 | 1 |
| 15 | LYZ | TTCAGATCAATAGCCGCTAC | 439 | 2 |
| 16 | HIF1A | CCTCACACGCAAATAGCTGA | 70 | 1 |
| 17 | ACYP1 | TTTCATAATCCACTGATATC | 38 | 3 |
| 18 | DRD1 | ATTGATACTCACCGTCTCTA | 28 | 3 |
| 19 | USP17L11 | CACCAAGTGCTCGTCCAACT | 26 | 3 |
| 20 | PCID2 | ACTTGTTGACACTTCTCCTC | 14 | 3 |
| 21 | DBP | ACGTACTCCACATCGCCGAA | 13 | 3 |
| 22 | ZNF12 | TTCCTACTTCAGAGCTATCC | 8 | 3 |
