# Supplementary material for: Genome-scale CRISPR-Cas9 knockout screening in gastrointestinal stromal tumor with Imatinib resistance
Source: Mol Cancer. 2018 Aug 13;17:121. doi: 10.1186/s12943-018-0865-2 (PMC6090611; doi:10.1186/s12943-018-0865-2)
Supplement: Supplementary file 7 — Table S5. Detailed information of GO analysis for the selected 20 genes. (DOCX 13 kb) [file 12943_2018_865_MOESM7_ESM.docx]

Table S5. Detailed information of GO analysis for the selected 20 genes

| GO Term | Description | Associated genes |
| --- | --- | --- |
| Biological process | response to light stimulus | PKM/PPP1CB/DRD1/TCF12/ZFP36 |
|  | associative learning | PKM/TCF12/ZFP36 |
|  | regulation of thymocyte apoptotic process | PPP1CB/ZFP36 |
|  | response to radiation | PKM/PPP1CB/DRD1/TCF12/ZFP36 |
|  | thymocyte apoptotic process | PPP1CB/ZFP36 |
|  | entrainment of circadian clock by photoperiod | PPP1CB/DRD1 |
|  | positive regulation of pri-miRNA transcription from RNA polymerase II promoter | TCF12/ZFP36 |
|  | photoperiodism | PPP1CB/DRD1 |
|  | positive regulation of transcription from RNA polymerase II promoter in response to stress | PPP1CB/DRD1 |
|  | entrainment of circadian clock | PPP1CB/DRD1 |
|  | regulation of hemopoiesis | DBP/PCID2/TCF12/ZFP36 |
|  | learning | PKM/TCF12/ZFP36 |
|  | regulation of pri-miRNA transcription from RNA polymerase II promoter | TCF12/ZFP36 |
|  | single-organism carbohydrate catabolic process | DRD1/HIF1A/ZFP36 |
|  | response to glucocorticoid | DBP/NR3C1/TCF12 |
|  | pri-miRNA transcription from RNA polymerase II promoter | TCF12/ZFP36 |
|  | carbohydrate catabolic process | DRD1/HIF1A/ZFP36 |
|  | regulation of T cell apoptotic process | PPP1CB/ZFP36 |
|  | response to corticosteroid | DBP/NR3C1/TCF12 |
|  | positive regulation of hemopoiesis | PCID2/TCF12/ZFP36 |
|  | regulation of erythrocyte differentiation | DBP/ZFP36 |
| Cellular component | RNA polymerase II transcription factor complex | PPP1CB/ZFP36/FOS |
|  | transcription factor complex | PPP1CB/TCF12/ZFP36/FOS |
|  | nuclear transcription factor complex | PPP1CB/ZFP36/FOS |
| Molecular function | transcriptional activator activity, RNA polymerase II transcription regulatory region sequence-specific binding | PPP1CB/NR3C1/TCF12/ZFP36/TP53/FOS |
|  | transcriptional activator activity, RNA polymerase II core promoter proximal region sequence-specific binding | NR3C1/TCF12/ZFP36/TP53/FOS |
|  | transcription factor activity, RNA polymerase II core promoter proximal region sequence-specific binding | NR3C1/TCF12/ZFP36/TP53/FOS |
|  | histone acetyltransferase binding | PPP1CB/ZFP36 |
|  | protein heterodimerization activity | PPP1CB/TCF12/ZFP36/FOS |
|  | transcription factor binding | PPP1CB/TCF12/ZFP36/FOS |
