# Supplementary material for: Genome-scale CRISPR-Cas9 knockout screening in gastrointestinal stromal tumor with Imatinib resistance
Source: Mol Cancer. 2018 Aug 13;17:121. doi: 10.1186/s12943-018-0865-2 (PMC6090611; doi:10.1186/s12943-018-0865-2)
Supplement: Supplementary file 9 — Table S6. KEGG pathway analysis of candidate genes. (DOCX 14 kb) [file 12943_2018_865_MOESM9_ESM.docx]

Table S6. KEGG pathway analysis of candidate genes

| Gene symbols | Pathway description |
| --- | --- |
| SOCS6 | Prolactin signaling pathway, JAK-STAT signaling pathway |
| ZFP36 | HTLV-I infection |
| TP53 | HTLV-I infection, Thyroid cancer, Bladder cancer, Amyotrophic lateral sclerosis (ALS), Endometrial cancer, Basal cell carcinoma, Non-small cell lung cancer, Colorectal cancer, Glioma, Pancreatic cancer, Central carbon metabolism in cancer, p53 signaling pathway, Melanoma, Chronic myeloid leukemia, Platinum drug resistance, Small cell lung cancer, Prostate cancer, Longevity regulating pathway, Endocrine resistance, Thyroid hormone signaling pathway, Neurotrophin signaling pathway, Sphingolipid signaling pathway, Cell cycle, Hepatitis C, Measles, Apoptosis, Wnt signaling pathway, Hepatitis B |
| ACYP1 | Pyruvate metabolism |
| DRD1 | Neuroactive ligand-receptor interaction, Cocaine addiction, Amphetamine addiction, Gap junction, Morphine addiction, Dopaminergic synapse, Parkinson's disease |
| NR3C1 | Neuroactive ligand-receptor interaction |
| ZNF12 | / |
| DBP | / |
| TCF12 | / |
